# Supplementary figures and images for: Testosterone pulses paired with a location induce a place preference to the nest of a monogamous mouse under field conditions
Source: eLife. 2022 Mar 30;11:e65820. doi: 10.7554/eLife.65820 (PMC9023057; doi:10.7554/eLife.65820)

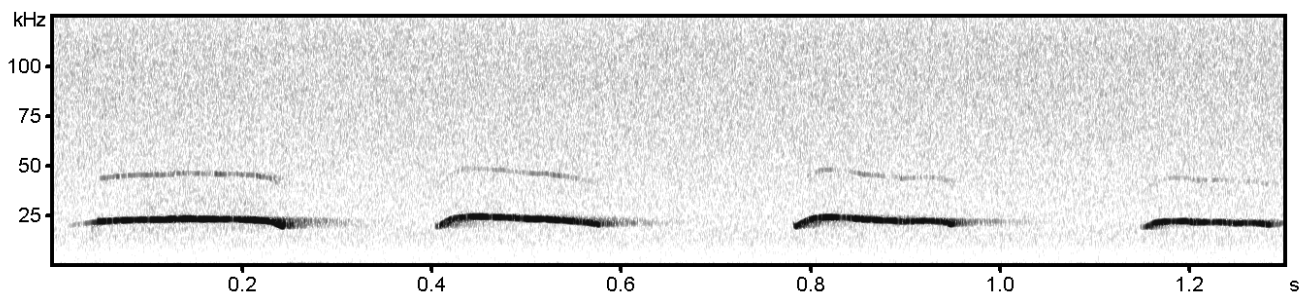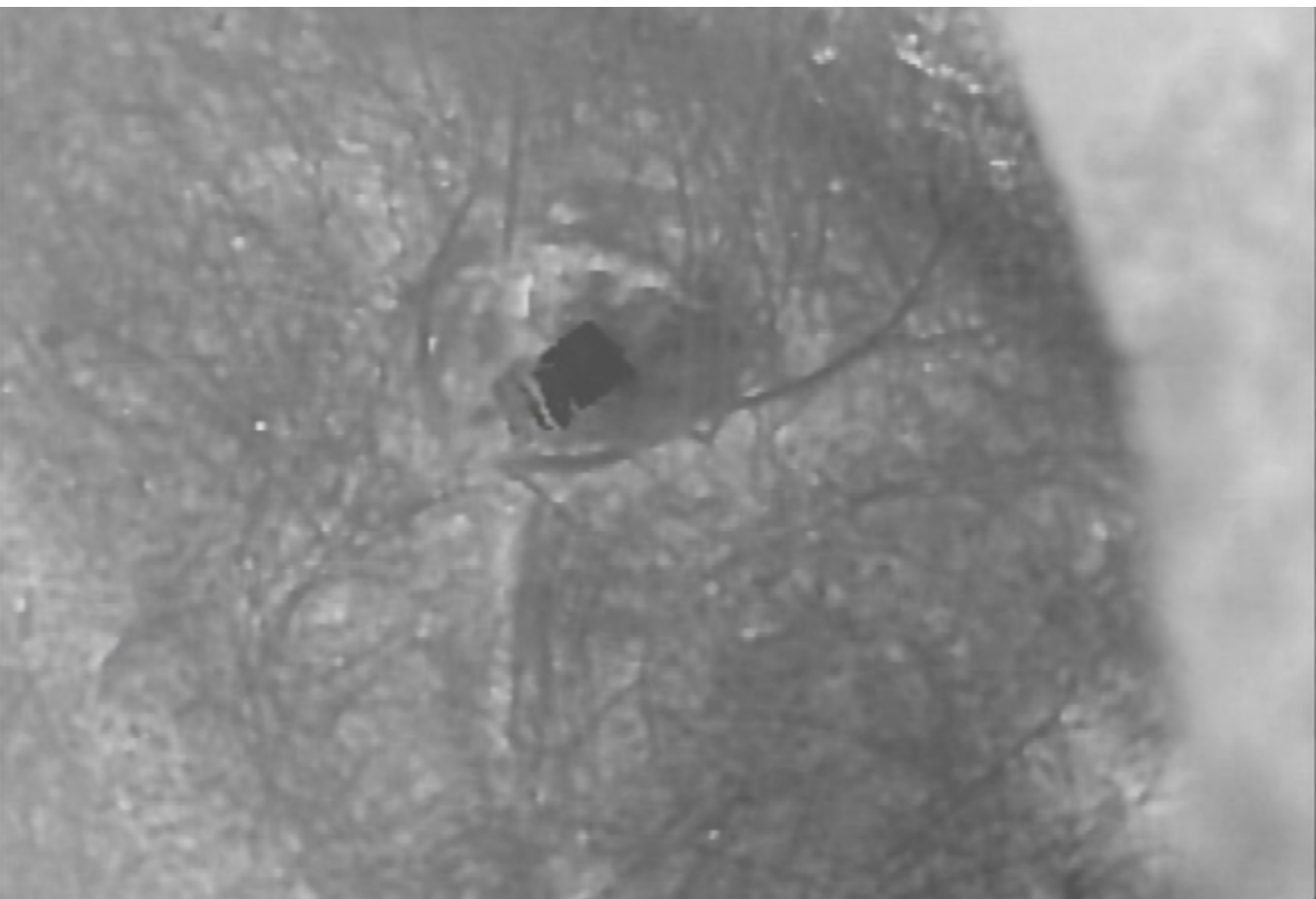

Supplement: Video 1—source data 1. [file elife-65820.zip › Source Data File/Video 2.pdf]
