# Supplementary material for: Testosterone pulses paired with a location induce a place preference to the nest of a monogamous mouse under field conditions
Source: eLife. 2022 Mar 30;11:e65820. doi: 10.7554/eLife.65820 (PMC9023057; doi:10.7554/eLife.65820)
Supplement: Supplementary file 1. [file elife-65820-supp1.docx]

**Supplemental** **File 1A**. Descriptive statistics for controls (C) and T-injected (T) male California mice including proportion of time spent at the nest with and without pups, proportion of time at the nest based on season, body mass (grams), number of nights required to administer three injections (days), and proportion of time at the nest based on recording night after the last injection. N represents the number of individuals (and not number of sampling nights).

**Supplemental** **File 1B**. Descriptive statistics for controls (C) and T-injected (T) female mice including proportion of time spent at the nest with and without pups, proportion of time at the nest based on season, body mass (grams), and proportion of time at the nest based on recording night after the last injection. N represents the number of individuals (and not the number of sampling nights).

**Supplemental** **File 1C**. Descriptive statistics for controls (C) and T-injected (T) for the time that both members of the pair were at the nest. N represents the number of individuals (and not the number of sampling nights).

**Supplemental** **File 1D**. Descriptive statistics are given for number of USVs produced at the nest, presence of pups at the nest, season, body mass (grams), number of nights required to administer three injections (days), and recording night after the last injection by treatment type. Each male received three T (n=14) or saline/control (n=12) injections at the nest. After the final injection we recorded USVs at the nest for three consecutive nights. For the first 5 variables, “n” in the table includes three data points for each pair (representing three nights). For the last variable, “n” represents the number of pairs.

**Supplemental** **File 1E.** The number of total USVs produced based on call type and the distance between the members of a pair. Distance was classified into three categories (apart: >2m; together: < 1m; intermediate: 1-2m apart).

**Supplemental** **File 1F**. Descriptive statistics and results from the Wilcoxon rank sum test for the comparison of USV proportion by type and treatment produced at the nest. Each male received three T (n=14) or C (n=12) injections at the nest. After the final injection, we recorded USVs at the nest for three consecutive nights. Alpha values of p <0.05 are in **bold**. N represents the number of individuals.

**Supplemental** **File 1G.** Descriptive statistics on spectral characteristics of male calls are given for the first call in the sequence for 1, 2-, 3- and 4SVs produced by males (T: n=12 and C: n=6).
