# Supplementary material for: Testosterone pulses paired with a location induce a place preference to the nest of a monogamous mouse under field conditions
Source: eLife. 2022 Mar 30;11:e65820. doi: 10.7554/eLife.65820 (PMC9023057; doi:10.7554/eLife.65820)
Supplement: Transparent reporting form [file elife-65820-transrepform1.pdf]

Westbrook Centre, Milton Road
Cambridge CB4 1YG
UK

P 01223 855340
W elifesciences.org
T @elife

eLife's transparent reporting form

We encourage authors to provide detailed information *within their submission* to facilitate the interpretation and replication of experiments. Authors can upload supporting documentation to indicate the use of appropriate reporting guidelines for health-related research (see EQUATOR Network), life science research (see the BioSharing Information Resource), or the ARRIVE guidelines for reporting work involving animal research. Where applicable, authors should refer to any relevant reporting standards documents in this form.

If you have any questions, please consult our Journal Policies and/or contact us: editorial@elifesciences.org.

Sample-size estimation

- You should state whether an appropriate sample size was computed when the study was being designed
- You should state the statistical method of sample size computation and any required assumptions
- If no explicit power analysis was used, you should describe how you decided what sample (replicate) size (number) to use

Please outline where this information can be found within the submission (e.g., sections or figure legends), or explain why this information doesn't apply to your submission:

Based on the length of our field season, we calculated the maximum number of mouse pairs we could feasibly study (record). For our final datasets we obtained the maximum number of pairs for each treatment group. Our sample size numbers are similar to the sample sizes of similar published behavioral studies. The final sample size numbers are above the minimum replicate requirement necessary for statistical analysis. We did not conduct an explicit power analysis.

Replicates

- You should report how often each experiment was performed
- You should include a definition of biological versus technical replication
- The data obtained should be provided and sufficient information should be provided to indicate the number of independent biological and/or technical replicates
- If you encountered any outliers, you should describe how these were handled
- Criteria for exclusion/inclusion of data should be clearly stated
- High-throughput sequence data should be uploaded before submission, with a private link for reviewers provided (these are available from both GEO and ArrayExpress)

Please outline where this information can be found within the submission (e.g., sections or figure legends), or explain why this information doesn't apply to your submission:

Our experiments were conducted on 28 distinct pairs (line 443). Time spent at the nest was recorded for 21 males consisting of 63 nights and for 23 females for 69 nights (line 484-486). Vocalization data were recorded from 26 pairs for 78 nights (line 510-512). For our analysis we used all the individuals we collected data from and no outliers were removed.

Westbrook Centre, Milton Road
Cambridge CB4 1YG
UK

P 01223 855340
W elifesciences.org
T @elife

Statistical reporting

- Statistical analysis methods should be described and justified
- Raw data should be presented in figures whenever informative to do so (typically when N per group is less than 10)
- For each experiment, you should identify the statistical tests used, exact values of N, definitions of center, methods of multiple test correction, and dispersion and precision measures (e.g., mean, median, SD, SEM, confidence intervals; and, for the major substantive results, a measure of effect size (e.g., Pearson's r, Cohen's d)
- Report exact p-values wherever possible alongside the summary statistics and 95% confidence intervals. These should be reported for all key questions and not only when the p-value is less than 0.05.

Please outline where this information can be found within the submission (e.g., sections or figure legends), or explain why this information doesn't apply to your submission:

Statistical analyses were described in detail on Statistical Analysis (line 537 to 566) of the Methods section. Appropriate programs, statistical packages and published papers were citied within the statistical analysis section. We fitted Generalized Linear Mixed Models (GLMM) with individual as the random term and treatment type as the fixed term. To compare call types, we used a Wilcoxon Rank Sum test. Lastly, for call characteristics, we conducted a principal component analysis for all the frequency variables prior to running a GLMM. Data for behavioral analyses are presented in figures 2-5 and details including mean, medial, maximum, minimum were reported in the supplementary tables 1-6.

(For large datasets, or papers with a very large number of statistical tests, you may upload a single table file with tests, Ns, etc., with reference to sections in the manuscript.)

Group allocation

- Indicate how samples were allocated into experimental groups (in the case of clinical studies, please specify allocation to treatment method); if randomization was used, please also state if restricted randomization was applied
- Indicate if masking was used during group allocation, data collection and/or data analysis

Please outline where this information can be found within the submission (e.g., sections or figure legends), or explain why this information doesn't apply to your submission:

Pairs were randomly assigned to a group by a third party with the requirement that equal number of pairs were assigned to each group. To avoid unconscious biases, all individual behaviors were analyzed prior to disclosing the treatment information.

Additional data files ("source data")

- We encourage you to upload relevant additional data files, such as numerical data that are represented as a graph in a figure, or as a summary table
- Where provided, these should be in the most useful format, and they can be uploaded as "Source data" files linked to a main figure or table
- Include model definition files including the full list of parameters used
- Include code used for data analysis (e.g., R, MatLab)
- Avoid stating that data files are "available upon request"

Please indicate the figures or tables for which source data files have been provided:

Source data are uploaded and available on Open Science Frameworks.
Petric, Radmila. 2021. "T-Pulses at the Nest." OSF. osf.io/qknze.
DOI 10.17605/OSF.IO/QKNZE

Data are available for Figures 2-5 and Tables 1-6.
